# A Comparison of ICU Mortality Scoring Systems Applied to COVID-19

**DOI:** 10.7759/cureus.35423

**Published:** 2023-02-24

**Authors:** Muhammad Monk, Jordan Torres, Kimberly Vickery, Gnananandh Jayaraman, Siva T Sarva, Ramesh Kesavan

**Affiliations:** 1 Internal Medicine, HCA Houston Kingwood/University of Houston College of Medicine, Kingwood, USA; 2 Internal Medicine, Univeristy of Houston/HCA Healthcare Kingwood, Houston, USA; 3 Medical Education and Simulation, HCA Healthcare, Brentwood, USA; 4 Pulmonary and Critical Care Medicine, HCA Houston Kingwood, Kingwood, USA; 5 Pulmonary and Critical Care Medicine, HCA Houston Kingwood/University of Houston College of Medicine, Houston, USA

**Keywords:** isaric 4c score, sequential organ failure assessment (sofa), acute physiology and chronic health evaluation (apache) ii, simplified acute physiology score (saps) ii, risk stratification, intensive care unit, corona virus mortality scores, covid-19

## Abstract

Background

Over the past three years, COVID-19 has been a major source of mortality in intensive care units around the world. Many scoring systems have been developed to estimate mortality in critically ill patients. Our intent with this study was to compare the efficacy of these systems when applied to COVID-19.

Methods

The was a multicenter, retrospective cohort study of critically ill patients with COVID-19 admitted to 16 hospitals in Texas from February 2020 to March 2022. The Simplified Acute Physiology Score (SAPS) II, Acute Physiology and Chronic Health Evaluation (APACHE) II, Sequential Organ Failure Assessment (SOFA) score, and 4C Mortality scores were calculated on the initial day of ICU admission. Primary endpoints were all-cause mortality, ICU length of stay, and hospital length of stay.

Results

Initially, 62,881 patient encounters were assessed, and the cohort of 292 was selected based on the inclusion of the requisite values for each of the scoring systems. The median age was 56 +/- 14.93 years and 61% of patients were male. Mortality was defined as patients who expired or were discharged to hospice and was 78%. The different scoring systems were compared using logistic regression, receiver operating characteristic (ROC) curve, and area under the ROC curve (AUC) analysis to compare the accuracy of prediction of the mortality and length of stay. The multivariate analysis showed that SOFA, APACHE II, SAPS II, and 4C scores were all significant predictors of mortality. The SOFA score had the highest AUC, though the confidence intervals for all of the models overlap therefore one model could not be considered superior to any of the others. Linear regression was performed to evaluate the models’ ability to predict ICU and hospital length of stay, and none of the tested systems were found to be significant predictors of length of stay.

Conclusion

The SOFA, APACHE II, ISARIC 4-C, and SAPS II scores all accurately predicted mortality in critically ill patients with COVID-19. The SOFA score trended to perform the best.

## Introduction

The COVID-19 pandemic has resulted in 664 million infections and more than 6.7 million mortalities as of January 2023. Due to the novelty of the disease and high virulence in patients with comorbidities, risk stratification and prognostication of outcomes proved to be a challenge [1].

Common mortality prediction scores used in intensive care settings include the Simplified Acute Physiology Score (SAPS) II, Acute Physiology and Chronic Health Evaluation (APACHE) II, and the Sequential Organ Failure Assessment (SOFA) [2-4] and have been used for decades and have been externally validated with several studies [5-7]. The ISARIC 4-C score [8] was designed in November 2020 specifically for COVID-19 and has not achieved widespread use despite external validation [9,10]. Our study looked to compare the efficacy of traditional scores and the 4C score with regard to all-cause mortality and ICU length of stay.

## Materials and methods

This was a retrospective cohort study of patients that were admitted to the ICU between February 2020 to March 2022 in 16 hospitals in South Texas with the approval of the IRB of the HCA Gulf Coast Division (Case Number: 2022-357). The collection of data took place by extracting the billing data from patients admitted to the participating hospitals during the study period. No patient-identifying information was collected as part of the study Inclusion criteria consisted of adults admitted to the ICU with a positive rapid antigen or PCR for COVID-19, and patients who had all laboratory values to calculate each mortality score on the day of ICU admission. Patients admitted from hospice were excluded.

The data collected included the history of cirrhosis, heart failure Class IV, chronic lung disease, or ESRD; age, heart rate, systolic blood pressure, MAP, respiratory rate, temperature, sodium, potassium, creatinine, presence of acute renal failure, hematocrit, leukocyte count, GCS, FiO_2_, presence of mechanical ventilation, platelets, bilirubin, administration of vasopressors, creatinine, urine output, CRP, and oxygen saturation on room air.

The cohort was separated into survivors and non-survivors (those who expired or were discharged to hospice). The measured scoring systems were calculated for each patient in SAS. Likelihood of survival, ICU length of stay, and hospital length of stay were analyzed in SPSS.

**Figure 1:**
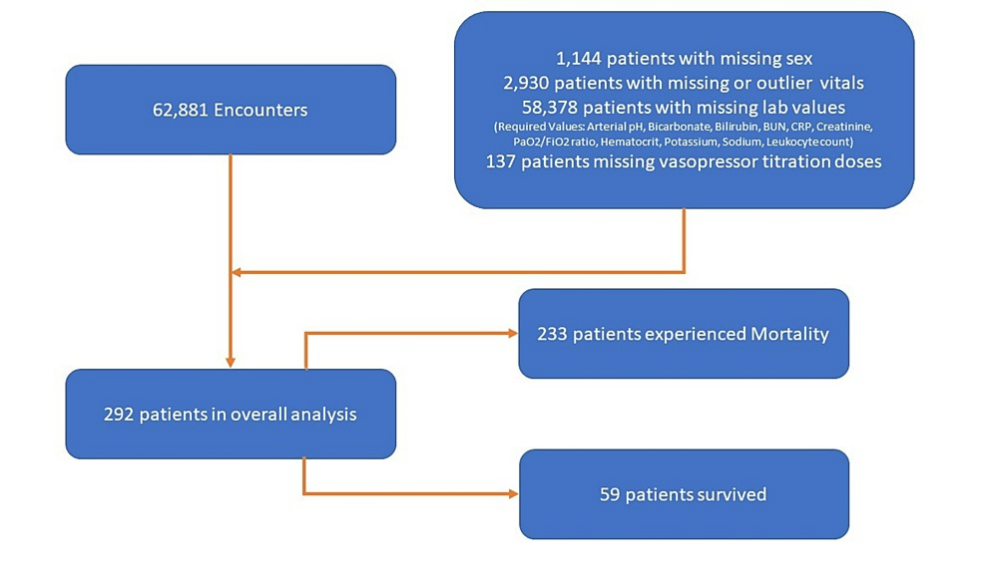
Study flow diagram BUN: Blood Urea Nitrogen, CRP: C-reactive Protein, PaO2: Partial Pressure of arterial oxygen, FiO2: Fraction of Inspired Oxygen

A total of 62,881 patients were admitted to the ICU during the study period, and 292 patients were included in the analysis (Figure 1). The median age of the cohort was 56 +/- 14.93 years and 61% of patients were male. Patients missing required values for scoring systems were excluded and further information can be found below (Figure 1).

## Results

Of the 292 patients included, 59 patients survived and 233 expired or were discharged to hospice (79.7% mortality). Logistic regression was used to predict the likelihood of mortality vs survival. All the scoring systems were significant in predicting mortality (Table 1). Logistic regression in the standardized model compared the systems to each other. A one-standard unit increase in SOFA had the largest increase in the likelihood of mortality among the four systems; however, the confidence intervals overlap indicating that no one model is any better than the others at predicting mortality (Table 1).

**Table 1:** Analysis of scoring systems SOFA: Sequential Organ Failure Assessment, 4C: ISARIC-4C Mortality Score, SAPS: Simplified Acute Physiology Score, APACHE II: Acute Physiology and Chronic Health Evaluation, B: Beta Coefficient For Linear Regression

Standardized Likelihood of Mortality by Scoring System			
Scoring System	Odds Ratio	95% Confidence Interval	P-value
SOFA	1.964	1.449-2.661	<0.001
4C	1.649	1.226-2.217	<0.001
SAPS	1.550	1.161-2.069	0.003
APACHE II	1.588	1.192-2.116	0.002
Hospital Length of Stay by Scoring System			
Scoring System	B	95% Confidence Interval	P-Value
SOFA	0.087	-0.682, 0.857	0.823
4C	-0.379	-0.964, 0.206	0.203
SAPS	0.027	-0.120, 0.173	0.721
APACHE II	0.192	-0.058, 0.443	0.132
ICU Length of Stay by Scoring System			
Scoring System	B	95% Confidence Interval	P-Value
SOFA	0.040	-0.524, 0.604	0.889
4C	-0.224	-0.670, 0.183	0.262
SAPS	0.082	-0.024, 0.188	0.127
APACHE II	0.221	0.040, 0.402	0.017

**Figure 2:**
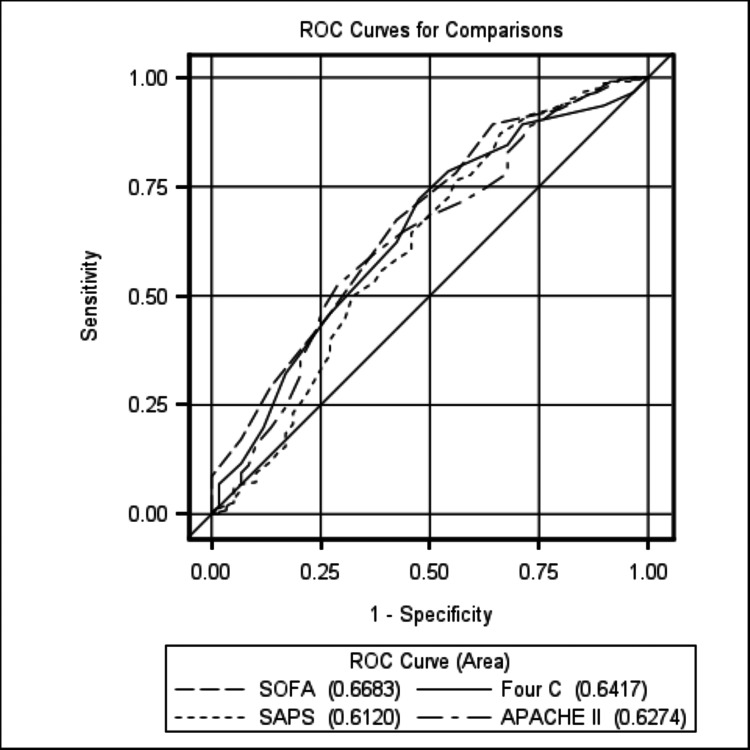
ROC-AUC analysis ROC - Receiver operating characteristic curve, AUC - area under the ROC curve

Receiver operating characteristic (ROC) curve and area under the ROC curve (AUC) analysis were performed for all of the scoring systems studied (Figure 2). Each of the systems has an AUC above 0.5 showing each model is a better predictor than random chance. Although SOFA had the highest AUC, the systems’ 95% confidence intervals overlapped, suggesting that no one model is any better at predicting mortality than the others.

Linear regression measured the models’ ability to predict ICU and hospital length of stay. Analysis was performed on 289 patients, three patients with outlier length of stays were removed. None of the models were found to be accurate predictors for either hospital or ICU length of stay. APACHE II was found to be significant at measuring ICU length of stay; however, the r-square value was 0.021 indicating minimal utility in practice. None of the models were found to be significant predictors of hospital length of stay.

## Discussion

The results showed each of the models was an accurate predictor of mortality in critically ill patients infected with COVID-19. The SOFA score trended to have the highest per-unit increase in the likelihood of mortality across the four models but was not significant. Based on these results any of the studied models could be used as independent predictors of mortality in the study population.

None of the scoring systems were accurate predictors of ICU or hospital length of stay. It should be noted that none of these were designed for this purpose. The length of stay may also be artificially decreased, as the population had a high rate of mortality.

The study shows a higher mortality rate compared to prior published studies [11-15]. Patients with all of the required lab values to calculate each of the four scores could have been more critically ill, potentially resulting in bias. Over 62,000 patients were screened for inclusion in the study and the overwhelming majority of those excluded were removed due to missing laboratory values required to complete all of the tested scoring systems. Given these requirements, the cohort of included patients was smaller than expected, though the results were sufficiently powered to demonstrate statistical significance. A larger cohort of patients in a prospective study could be beneficial in further determining the utility of the tested scoring systems.

The scores were calculated on the first day of ICU admission. Notably, the ISARIC 4C score was designed to be calculated in the emergency department, therefore was not used in its intended function in this study. Despite this, it was found to be a good predictor of mortality and may have clinical utility in practice. Notably, with the exception of the 4C score, these systems were not designed specifically to estimate mortality in COVID-19. Attributable mortality from COVID-19 could not be measured as part of this study.

A Lithuanian study [11] in 2020 found the APACHE II score and 4C score to be the most accurate when compared to SOFA and SAPS II in COVID-19 patients. Similarly, a Belgian study [12] from 2021 concluded APACHE II outperformed the SOFA score, with the APACHE IV score [13] (not tested in our study) performing the best overall. Another study from Iran [14] published in 2022 showed the daily SOFA score to be superior to the APACHE II when evaluating mortality. Two of the three examined external validation studies [5,6] for the scoring systems conducted prior to the discovery of COVID-19 showed any of the systems could be used reliably, with the third [7] showing better performance for the SAPS II. Based on these previously reported results, there is an absence of a consensus clear-cut scoring system of choice It is likely more testing of these systems is needed in order to establish whether any given score performs better than the others, particularly in patients with COVID-19. It should also be noted that the APACHE II score requires additional lab values compared to the other scores that may not always be routinely collected, thereby somewhat limiting its generalizability and daily use in clinical practices.

To our knowledge, our study is the first United States based study evaluating the performance of mortality scores with regard to critically ill patients with COVID-19. One previous study [15] was conducted to evaluate the efficacy of the APACHE II and qSOFA scores for prognostication of ICU admission; however, mortality was not used as a primary endpoint. That study also used the qSOFA score developed in 2016 as part of the Sepsis-3 guidelines [16], a score that was not tested in this study. Our study is unique in that all relevant labs for each mortality score were drawn on day 1 of ICU admission in order to ensure accurate calculations. Our study included a large initial pool of COVID-19 patients from multiple centers so that a large number of patients with all laboratory values could be included for further analysis. The other studies evaluating COVID-19 patients were all conducted at single centers, and it was unclear how missing lab values to calculate scores were addressed. The difference in inclusion criteria of patients with missing laboratory values could have contributed to the difference in results. To understand the utility of the individual scoring systems for COVID-19 positive patients, further prospective studies may be useful.

## Conclusions

COVID-19 has proven to be an unpredictable disease. It is important to be able to determine the severity of illness in the critical care setting. This study showed the SOFA, APACHE II, ISARIC 4-C, and SAPS II scores all accurately predicted mortality in critically ill patients with COVID-19. This was in spite of different virus strains and evolving treatment regimens for COVID-19. The SOFA score trended to perform the best however was not statistically significant. In the absence of a consensus best mortality scoring system, further prospective studies should be performed to determine the effectiveness of these scores and if the SOFA score is the most accurate predictor of mortality.

## References

[1] Ejaz H, Alsrhani A, Zafar A (2020). COVID-19 and comorbidities: deleterious impact on infected patients. J Infect Public Health.

[2] Vincent JL, Moreno R, Takala J (1996). The SOFA (Sepsis-related Organ Failure Assessment) score to describe organ dysfunction/failure. On behalf of the Working Group on Sepsis-Related Problems of the European Society of Intensive Care Medicine. Intensive Care Med.

[3] Knaus WA, Draper EA, Wagner DP, Zimmerman JE (1985). APACHE II: a severity of disease classification system. Crit Care Med.

[4] Le Gall JR, Lemeshow S, Saulnier F (1993). A new Simplified Acute Physiology Score (SAPS II) based on a European/North American multicenter study. JAMA.

[5] Czajka S, Ziębińska K, Marczenko K, Posmyk B, Szczepańska AJ, Krzych ŁJ (2020). Validation of APACHE II, APACHE III and SAPS II scores in in-hospital and one year mortality prediction in a mixed intensive care unit in Poland: a cohort study. BMC Anesthesiol.

[6] Granholm A, Møller MH, Krag M, Perner A, Hjortrup PB (2016). Predictive performance of the Simplified Acute Physiology Score (SAPS) II and the initial Sequential Organ Failure Assessment (SOFA) score in acutely ill intensive care patients: post-hoc analyses of the SUP-ICU inception cohort study. PLoS One.

[7] Godinjak A, Iglica A, Rama A, Tančica I, Jusufović S, Ajanović A, Kukuljac A (2016). Predictive value of SAPS II and APACHE II scoring systems for patient outcome in a medical intensive care unit. Acta Med Acad.

[8] Knight SR, Ho A, Pius R (2020). Risk stratification of patients admitted to hospital with covid-19 using the ISARIC WHO Clinical Characterisation Protocol: development and validation of the 4C Mortality Score. BMJ.

[9] Wirth A, Goetschi A, Held U, Sendoel A, Stuessi-Helbling M, Huber LC (2022). External validation of the modified 4C deterioration model and 4C mortality score for COVID-19 patients in a Swiss tertiary hospital. Diagnostics (Basel).

[10] Knight SR, Gupta RK, Ho A (2022). Prospective validation of the 4C prognostic models for adults hospitalised with COVID-19 using the ISARIC WHO Clinical Characterisation Protocol. Thorax.

[11] Vicka V, Januskeviciute E, Miskinyte S (2021). Comparison of mortality risk evaluation tools efficacy in critically ill COVID-19 patients. BMC Infect Dis.

[12] Vandenbrande J, Verbrugge L, Bruckers L (2021). Validation of the Acute Physiology and Chronic Health Evaluation (APACHE) II and IV score in COVID-19 patients. Crit Care Res Pract.

[13] Zimmerman JE, Kramer AA, McNair DS, Malila FM (2006). Acute Physiology and Chronic Health Evaluation (APACHE) IV: hospital mortality assessment for today's critically ill patients. Crit Care Med.

[14] Beigmohammadi MT, Amoozadeh L, Rezaei Motlagh F (2022). Mortality predictive value of APACHE II and SOFA scores in COVID-19 patients in the intensive care unit. Can Respir J.

[15] Wilfong EM, Lovly CM, Gillaspie EA (2021). Severity of illness scores at presentation predict ICU admission and mortality in COVID-19. J Emerg Crit Care Med.

[16] Singer M, Deutschman CS, Seymour CW (2016). The Third International Consensus Definitions for sepsis and septic shock (Sepsis-3). JAMA.

